# Management of severe and rigid idiopathic scoliosis

**DOI:** 10.1007/s00590-015-1650-1

**Published:** 2015-06-02

**Authors:** Luis Eduardo Carelli Teixeira da Silva, Alderico Girão Campos de Barros, Gustavo Borges Laurindo de Azevedo

**Affiliations:** Center of Spine Diseases, Instituto Nacional de Traumatologia e Ortopedia - INTO, Av. Brasil 500, Rio de Janeiro, 20940-070 Brazil

**Keywords:** Scoliosis, Rigid, Severe, Idiopathic, Osteotomy

## Abstract

Frequently, severe idiopathic scoliosis patients are first seen in a spine centre after years of deformity evolution, presenting with large curves, severe rib hump, shoulder and trunk imbalance and cardiorespiratory complications related to neglected scoliosis. Severe rigid idiopathic scoliosis has <25 % of correction on bending films and major curve over 90°. Adequate mobilization of this type of deformity is necessary to achieve maximal correction, often requiring more extensive surgical intervention, with care taken to avoid clinical and neurological complications. Halo traction, internal temporary distraction, releases, osteotomies and apical vertebral resection are often used in combination to achieve optimal results. Indications must be tailored by surgeons considering resources, deformity characteristics and patient’s profile. Vertebral resection procedures may have potential neurological and clinical risks and should be one of the last treatment options performed by experienced surgical team. Neuromonitoring is essential during these procedures.

## Introduction

Idiopathic scoliosis when untreated or not treated properly may lead to severe complications related to curve progression. Delay in diagnosis and treatment, as well as aggressive patterns, can lead to severe idiopathic curves. Expert consensus has determined a maximum waiting time of 6 months for surgery in patients with adolescent idiopathic scoliosis (AIS) [1]. Recent studies have shown that this period may be too long for some patients, in whom a 3-month period may be the maximum acceptable wait time for surgery [2]. Patients who wait more than 3 months for idiopathic scoliosis surgery have higher probability of adverse events and revision surgery rates. The highest risks of adverse events due to prolonged wait times occur in patients who are skeletally immature and have larger curvatures of the spine. The feasibility of meeting an ideal access target has resource implications: Sufficient operating room time, spinal surgeons, hospitals and funding are necessary [3].

In developing countries, the ideal waiting time for scoliosis surgery is not a reality and the wait lists can reach more than 5 years [4]. Generally, severe idiopathic scoliosis patients are first seen in a spine centre after years of deformity evolution, presenting with large curves, severe rib hump, shoulder and trunk imbalance and cardiorespiratory complications related to neglected scoliosis.

Severe rigid idiopathic scoliosis has <25 % of correction on bending films and major curve over 90° [5]. Adequate mobilization of this type of deformity is necessary to achieve maximal correction, often requiring more extensive surgical intervention, with care taken to avoid clinical and neurological complications. Halo traction, internal temporary distraction, releases, osteotomies and apical vertebral resection are often used in combination to achieve optimal results. These techniques are often a component of extensive surgeries and require meticulous surgical planning that includes consideration of the curve location, magnitude and stiffness of curve and sagittal and coronal balance. Patient’s overall medical condition and ability to tolerate extensive procedures, surgeon’s skills and available resources will dictate management.

## Halo traction

Large stiff spinal deformities are challenging to treat, and their rapid or extensive correction is associated with increased neurological risk. Spine traction, internal or external, allows progressive improvement in spinal deformity, diminishing the stress on implants applied to the curve and giving the opportunity for easy and rapid neurological monitoring by clinical examination while patient is in traction [6]. External traction is applied with cranial halo and counter-traction through femur, pelvis or body weight (gravity). Internal traction is done through distraction rods anchored to the curve ends. External traction can be applied preoperatively, intraoperatively or in-between surgical times on staged procedures. The authors use halo-gravitational traction in severe scoliosis treatment, performing Ponte osteotomies on first approach, then applying increasing traction over a period of 2–4 weeks. After this traction period, the final correction is done, with other osteotomies or releases as needed [7].

## Temporary internal distraction

Temporary internal distraction has emerged as an alternative to external traction. It consists in inserting temporary internal rods to provide maximal longitudinal distraction over the area of greatest deformity. Indications for the technique include patients in whom greater mobility is desired, those with lumbar deformity and patients with contraindications to cervical traction. The technique involves a release over the rigid portion of the deformity and anchor placement near the intended upper and lower end vertebra of the curve [6, 8]. Careful distraction is performed under neuromonitoring and usually exceeds what is achieved in the preoperative traction films. If curve correction of approximately 50 % or more than 50° is not achieved with the first distraction procedure, an additional distraction procedure is performed 1–2 weeks after initial surgery [9, 10].

**Fig. 1:**
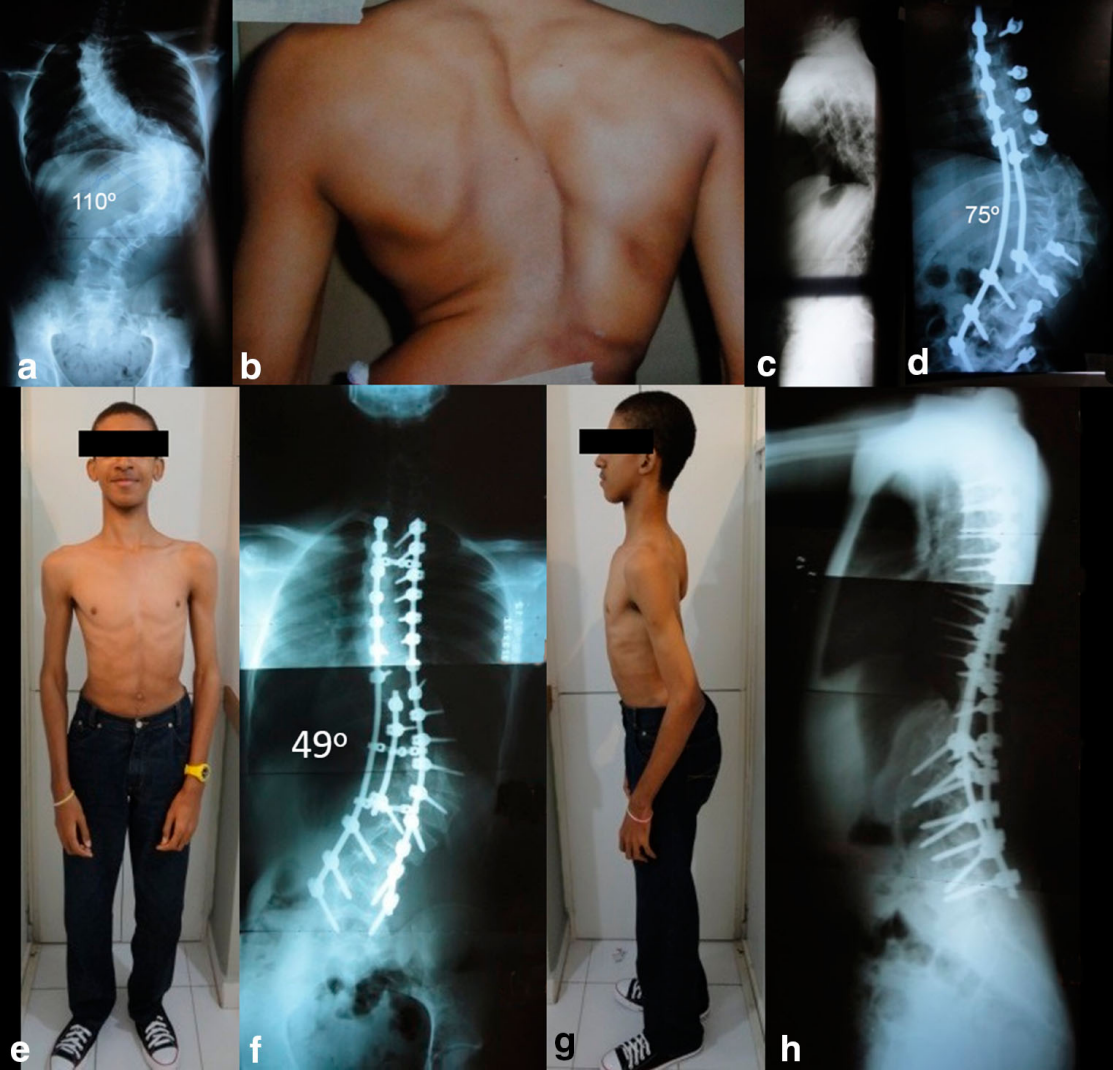
18-year-old male with severe rigid adolescent idiopathic scoliosis with 110° main thoracolumbar curve. Patient underwent a staged procedure. First, an internal distraction and posterior release followed by posterior spine fusion 1 week later. **a**–**c** Preoperative radiographical and clinical images. **d** PA radiograph after internal distraction. **e**–**h** Postoperative clinical and radiographical images

Temporary internal distraction can also be used in combined anterior–posterior procedures. This technique can allow up to 78 % correction of severe curves over 100° with less time spent in hospital [8]. A study comparing anterior and posterior vertebral column resection (VCR) versus anterior release with temporary distraction for severe and rigid scoliosis showed better corrective effects with anterior release with internal distraction than anterior or posterior VCR (75.6 vs. 67.7 %) [9]. These results show that internal distraction can be an alternative method for the treatment of severe scoliosis without the need of a more demanding osteotomy (Fig. 1).

## Anterior release

Anterior release may be performed in the thoracic and lumbar spine to allow flexibilization and correction of sagittal and coronal deformities. The anterior release and fusion are performed through either an endoscopic or open approach with similar results. Severe deformities course with anatomic changes on chest wall and spine making endoscopic approach impractical. Both approaches have negative impact on pulmonary function when compared to posterior-only approach [11, 12]. In the thoracic spine, the convex rib heads are resected and the discs and posterior annulus are removed, with release of the posterior longitudinal ligament. The convex inferior endplate is then resected with or without resection of the convex superior endplate. Anterior structural support is often indicated in the lumbar spine and at the thoracolumbar junction to avoid kyphosis. After anterior release and fusion, severe and rigid curves can be instrumented combined, anteriorly and posteriorly, with safe and effective tridimensional correction. The use of total pedicular constructs, with the improved segmental fixation and better ability to tridimensionally correct the AIS curves, have diminished the need for anterior approach in selected curves [13].

## Posterior extrapleural intervertebral space release (PEISR)

Anterior procedures whether done through an open thoracotomy or via an endoscopic approach have a detrimental impact on respiratory function [12, 14]. Chao Li et al. developed the concept of posterior extrapleural intervertebral space release. In this technique, the intervertebral discs and anterior longitudinal ligament are removed at two or three disc levels, both cranial and caudal to the apex to achieve adequate release through a posterior-only approach, thus not violating the thoracic cavity. This is a challenging but effective technique to treat severe and rigid spinal deformity. Most of the cases of severe and rigid AIS are long three-dimensional deformities that involve several vertebral levels, and a VCR does not affect the flexibility of the segments above and below the osteotomy, thus not correcting the three-dimensional deformity of the spinal column.

**Fig. 2:**
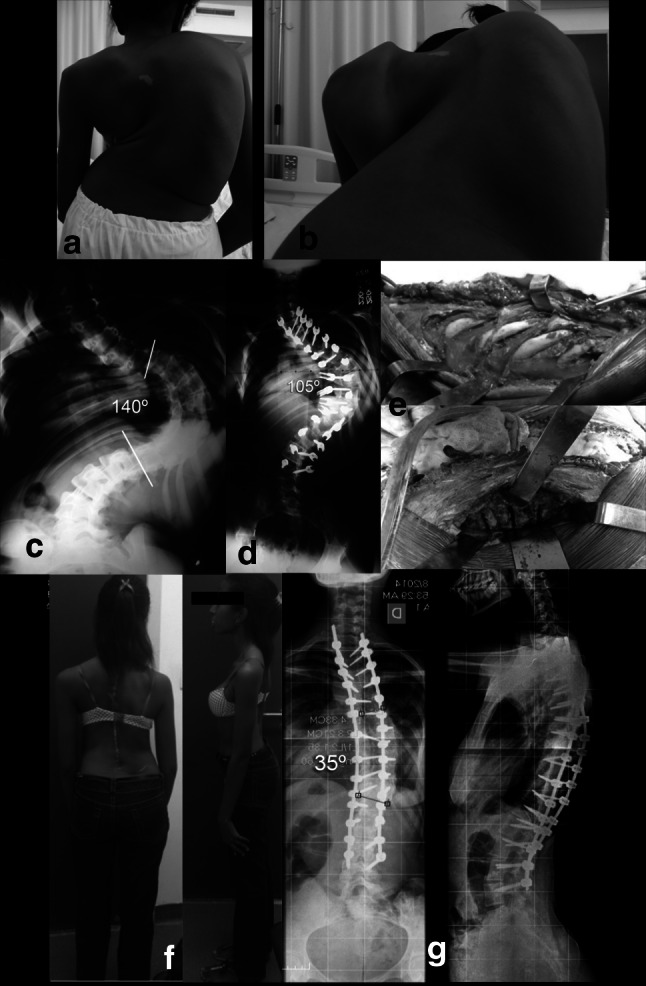
20-year-old patient with neglected AIS—severe curve of 140° treated with staged procedures. **a**, **b** Preoperative clinical pictures. **c** Preoperative PA radiograph. **d** Radiograph after posterior release and instrumentation and 2 weeks of traction. **e** Intraoperative pictures of PEISR technique. **f**, **g** Postoperative clinical and radiographical correction

PEISR can achieve similar results with less instability when compared to VCR, with theoretically less neurological risk [15] (Fig. 2).

## Posterior release

Severe scoliosis involves bony changes that cannot be corrected through the release of soft tissue alone. The choice of osteotomy used depends on the amount of correction needed, location of the deformity, the sagittal and coronal imbalance, patient’s condition and surgeon’s abilities [16]. The Smith–Petersen osteotomies (SPO) can be used for mobilization and correction of sagittal and coronal deformities. The SPO procedure lengthens the anterior column and closes the posterior column, resulting in a posterior shift of the gravity line. The spinous processes, ligament flavum, laminae and facet joints are excised [17]. Care should be taken to avoid pseudoarthrosis, because of the gap created in the disc space. Wide foraminotomies must be performed to avoid nerve root compression during the closing of the osteotomy. Open-wedge osteotomies should be avoided in the thoracic spine to prevent lengthening of the thecal sac and neurological risk. Ponte osteotomies consist of a posterior resection of superior and inferior facets, laminae, ligament flavum and spinous processes. Ponte osteotomy differs from SPO because there is no or minimum lengthening of the anterior column of the spine, being used safely in the thoracic spine. Wide removal of laminae is important to prevent spinal cord compression [18].

Pedicle subtraction osteotomy (PSO) is a dorsally based three-column closing-wedge osteotomy. The symmetrical use of this osteotomy promotes correction and mobilization of the deformity in the sagittal plane, resulting in 30°–35° of correction per level. Most of the PSOs are performed with the purpose of treating sagittal imbalance, but a significant coronal correction can also be achieved with an asymmetric osteotomy. A larger wedge resected on the convexity of the kyphoscoliotic deformity results in focal coronal correction [19]. The use of a transdiscal PSO approach that more closely resembles a VCR in terms of correction mechanics achieves a mean correction of lumbar lordosis of 40° with each osteotomy [20].

## Vertebral column resection

The procedure involves the resection of one or more vertebral levels and can be done through a combined anterior–posterior approach or a posterior-only approach. Combined anterior–posterior VCR is a technically demanding procedure with extensive operating times and high incidence of complications [21]. Posterior VCR has several advantages over anterior–posterior VCR: reduction in the total operating time and the amount of blood loss, better maintenance of spinal stability through-out the procedure, more reliable reconstruction of the spinal column enabling an immediate anterior structural support, less pulmonary morbidity and more effective correction of the deformity and the imbalance of the trunk [5, 22]. VCR is indicated for severe and rigid spinal deformities that cannot be corrected by osteotomies alone. The number of vertebral levels to be excised is based on the magnitude and rigidity of the curves. The instability created during the correction of the deformity can compromise the spinal cord by the rotational and shear forces. If the anterior gap created after closure of the osteotomy is larger than 5 mm, a cage or structural graft should be inserted to provide anterior column support without excessive shortening [5].

The VCR procedure allows for markedly clinical and radiographical correction. Sometimes, the radiographical correction does not reflect the clinical outcome in severe and rigid AIS patients and Cobb measures in these cases often under-represent the deformity correction and the benefit of the VCR [23]. This is highlighted by the significant improvement in the SRS subscores of self-image and satisfaction postoperatively. The complexity of the procedure, the nature of the spinal deformity and often-debilitated patients make complications very common, and this technique should remain one of the last resorts when no simpler method of spinal reconstruction will suffice [24].

## Rib hump treatment

Rib hump correction is highly related to patient satisfaction in scoliosis surgery. The modern thoracoplasty technique was developed by Howard Steel in 1983 [25]. Thoracoplasty promotes cosmetic improvement and provides additional source of autologous bone graft with low impairment in pulmonary function after 2 years of follow-up. Indications for thoracoplasty include prominence >15° on scoliometer at highest point of the deformity, curve flexibility of <20 % and curves >60° [26]. Even with the modern techniques of indirect and direct vertebral derotation, thoracoplasty still has its indications [27]. Despite being a very rewarding procedure and related to better levels of satisfaction after surgery in the self-image questionnaires, thoracoplasty is not a risk-free procedure and can lead to temporary pulmonar function impairment, chest wall pain, potential risk of chest drainage, flail chest and pseudoarthrosis [28].

**Fig. 3:**
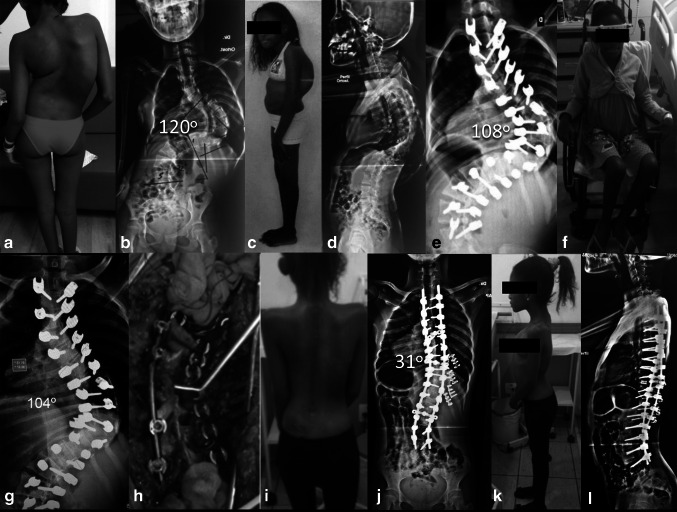
11-year-old female with severe rigid juvenile idiopathic scoliosis. Patient underwent preoperative halo-gravitational traction followed by staged procedures. **a**–**d** Preoperative clinical and radiographic images. **e**, **f** First, posterior release and instrumentation and postoperative halo-gravitational traction. **g** Radiograph showing poor correction with traction. **h**–**l** Second-stage posterior vertebral column resection and thoracoplasty reconstruction with internal osteosynthesis (TRIO)

Recently, believing in the benefits of osteosynthesis after the partial resection of rib, we developed a new strategy in the treatment of moderate and severe rib hump deformity for scoliosis patients. Thoracoplasty reconstruction with internal osteosynthesis (TRIO) consists of stabilization of rib stumps after partial costectomy using rib clips. Possible advantages of TRIO technique are better correction of rib prominence, lower postoperative impairment of pulmonary function, lower chest wall pain and less rib pseudoarthrosis [29] (Fig. 3).

## Conclusion

Halo traction, releases and osteotomies have proven to be highly efficacious in the treatment of severe and rigid idiopathic scoliosis. These techniques may be combined to achieve optimal correction. Indications must be tailored by surgeons considering resources, deformity characteristics and patient’s profile. Vertebral resection procedures may have potential neurological and clinical risks and should be one of the last treatment options and performed by experienced surgical team. Neuromonitoring is essential during these procedures.
